# Natural history and clinical response: “It’s the virus, stupid, or is it the host?”

**DOI:** 10.1186/1471-2334-12-S2-S6

**Published:** 2012-11-12

**Authors:** Stefania Nucara, Benedetto Caroleo, Vincenzo Guadagnino, Nicola Perrotti, Francesco Trapasso

**Affiliations:** 1 Unità Operativa di Genetica Medica, Policlinico Universitario Mater Domini, Università “Magna Græcia” di Catanzaro, Campus S. Venuta, 88100 Catanzaro, Italy; 2 Unità Operativa di Malattie Infettive, University Hospital Policlinico Mater Domini, Policlinico Universitario Mater Domini, Università “Magna Græcia” di Catanzaro, Campus S. Venuta, 88100 Catanzaro, Italy; 3 Dipartimento di Medicina Sperimentale e Clinica, Università “Magna Græcia” di Catanzaro, Campus S. Venuta, 88100 Catanzaro, Italy; 4 Dipartimento di Scienze della Salute, Università “Magna Græcia” di Catanzaro, Campus S. Venuta, 88100 Catanzaro, Italy

## Abstract

**Summary:**

A major goal of modern medicine is the application of personalized therapies, consisting of decisions and practices tailored to the individual patient. Information about genetic variants, either mutant or polymorphic, represents the basis for the development of this clinical approach. Recently, several independent genome-wide association studies (GWAS) have identified two single nucleotide polymorphisms (SNPs) on the *IL28B* locus associated with HCV containment, spontaneous clearance, treatment response, and disease progression. In this minireview we will concisely discuss some critical genetic concepts that may have possible implications for clinical decisions in the treatment of HCV infection.

## Introduction

Genome-wide association studies (GWAS) represent a powerful tool to identify genetic variants associated with complex traits, including either risk disease or drug response or susceptibility to adverse drug reactions [1,2]. The human genome is composed of over 3 billion base pairs; as single nucleotide polymorphisms (SNPs) are distributed across the genome approximately every 300 bp, it is estimated that the human genome roughly contains 10 million SNPs. The principle of GWAS, based on microarray technology, consists on the analysis of up to 1 million common SNPs occurring in >5% of the population (HapMap project [3]). Hundreds of GWAS have been performed in the attempt to link genetic variants to human disease; however, although many loci have been identified in many diseases, the role of tag SNPs in the causation of disease is still not clear. In fact, only in some cases these variations may account for an altered gene expression, altered mRNA processing or functional activity [4].

With 170 million people estimated infected worldwide, Hepatitis C Virus (HCV) remains a major health problem with personal, social, and economic implication [5,6]. Current treatment for patients chronically infected with HCV is based on the administration of PEG-IFN-alpha plus ribavirin. However, only less than 50% of patients experience viral eradication (SRV, or sustained viral response with absence of virus 24 weeks after therapy completion) [7]. Moreover, therapy is frequently complicated by side effects limiting the adherence to therapy [8]. Therefore, the identification of factors affecting response to conventional therapies, remains an important need. These considerations prompted several investigators to perform GWA studies aimed to the identification of genetic variants which could significantly account for the different drug response observed among HCV patients; in 2009, independent groups reported two common variants in the *IL28B* locus (rs12979860 CC or rs8099917 TT) in patients that were more likely to respond to the combination of PEG-IFN-alpha/ribavirin than patients with other *IL28B* variants [9-13].

## *The IL28B* gene and its product

The *IL28B* gene, which encodes for the cytokine IFN-λ3, is located on the chromosomal region mapped to 19q13 along in a cluster containing also *IL29* and *IL28A* genes, coding for the cytokines IFN-λ1 and IFN-λ2, respectively [14,15]. The cytokines encoded by these three genes can be induced by viral infection and they act by triggering the Jak-STAT (Janus kinase – Signal Transducer and Activator of Transcription) pathway [16], following to their interaction with a heterodimeric receptor, composed of IL10 and IL28 receptors (IL10R and IL28R, respectively) [14]. As a result, these cytokines are able to modulate antiviral activity through both innate and adaptive immune system pathways [17].

## *IL28B* polymorphisms and their effects

Recently, independent GWAS investigations pointed their attention to two polymorphisms lying on the *IL28B* locus [9-13]. The first of these studies was conducted on a large cohort (1,137 patients) of European-American, African-American and Hispanic individuals with HCV genotype 1 infection after 48 weeks of combined IFN-alpha/ribavirin [9]. Among the candidate SNPs, the homozygous CC variant of the SNP rs12979860, located 3 Kb upstream of the *IL28B* gene, was identified as favourable genotype, as patients bearing this polymorphism were twice as likely to achieve an SVR compared to patients with the genotype CT or TT following combinatorial therapy. Two other independent studies [10,11], also identified a number of other SNPs significantly associated with SVR, primarily rs8099917 in the *IL28B* locus (TT versus GT and GG genotypes) in two cohorts of Australian and Japanese patients, respectively. In a fourth GWAS, the analysis also included patients infected with HCV 2, 3, and 4 other that 1; this study also confirmed the association between rs8099917 genotype and progression to chronic HCV infection and response to treatment [13]. However, rs8099917 was a neighboring SNP in strong linkage disequilibrium with rs12979860 and regression modeling found that the latter was the strongest predictor of SVR among all candidate SNPs [9]. Finally, in another report, it was investigated whether the SNP rs12979860 could also predict spontaneous HCV clearance; interestingly, after genotyping 1,008 patients with acute HCV infection, it was assessed that patients bearing the rs12979860 CC genotype were more likely to spontaneously clear the virus compared to patients with rs12979860 CT or TT genotypes [12]. Although the advantageous effects of the rs12979860 CC genotype are not well understood, assays of *IL28B* expression in peripheral blood mononuclear cells through real-time quantitative PCR showed lower *IL28B* expression levels in patients carrying the minor alleles [11].

In summary, a number of SNPs around the *IL28B* gene locus were found across GWAS to be associated with treatment response. Although population racial distribution, sample sizes, clinical phenotypes as well as the SNPs represented on chips may account for the discrepancies observed in such studies, the important conclusion is that all of them have pointed to *IL28B* as a predictor of treatment response.

## Clinical implications of *IL28B* polymorphisms

The *IL28B* genotype test can be used to predict clinical response to PEG-IFN-alpha and ribavirin in HCV genotype 1 patients. The test result indicates whether the patient carries a rs12979860 CC, CT, or TT genotype; this approach is currently representing a commonly pursued practice for completing the diagnostic path of HCV patients. In fact, patients who have the *IL28B* CC genotype are more likely to have a SVR with PEG-IFN-alpha and ribavirin treatment, whereas patients who have the CT or TT genotype are more likely to be nonresponders. This additional information can help clinicians about decisions to ensure the best management of chronic hepatitis C patients.

The *IL28B* polymorphism test is quite simple, as it requires either whole blood or buccal swab; the genomic DNA extracted from these samples will be processed by polymerase chain reaction (PCR) performed on the genomic region carrying the rs12979860 SNP; the amplification product will be then investigated by direct sequencing, currently considered the gold standard for determining the rs12979860 polymorphism. Alternatively, to determine the presence of all three rs12979860 variants (CC, TT, CT), a faster real-time PCR assay has been developed [18]; also commercial tests are now available.

The important question is how *IL28B* genetic testing can be used to improve care for patients. Host *IL28B* genotype is the strongest pretreatment predictor of response through its effect on viral kinetics [19]. However, it has to be taken in consideration that even though the 20% of HCV patients bears the favourable rs12979860 CC genotype they do not experience SVR. Moreover, only 53% of rs12979860 CC show spontaneous HCV clearance versus 28% of CT plus TT genotypes [12]. From a genetic point of view, this discrepancy might be explained through the intrinsic limitations of GWAS. In fact, as GWAS currently use common haplotypes occurring in >5% of the population, rarer variants (<5%) may not be identified; moreover, GWAS do not take into account dynamic interactions between genes or between genes and environment [4]. Therefore, the characterization of HCV patients needs to be performed also taking in consideration clinical features including fibrosis, age, insulin resistance, viral load and race [20].

So, the scary question is: How does IL28B genotype knowledge really affect clinical decision? The question raises controversial answers. As mentioned earlier, the predictive role of the *IL28B* SNP is based on the combination of PEG-IFN-alpha plus ribavirin. Recently, important changes are occurring in the treatment of HCV patients; in fact, the use of the new direct antiviral agents including the protease inhibitors boceprevir and telaprevir in association with PEG-IFN-alpha and ribavirin is going to represent a new standard of care [21,22]. Initial data suggest that the favourable *IL28B* allele also predict response to triple therapy even though its predictive effect might not be as strong as for the PEG-IFN-alpha plus ribavirin therapy [23]. Knowledge of *IL28B* status is valuable not only for the patients (chance of recovery and reduction of treatment duration) but also for the health system (reduced cost of expensive therapies); also *IL28B* genotyping should be able to avoid triple therapy with protease inhibitors. However, on the other hand, it would be hard to give up a conventional therapy just because of the favourable *IL28B* genotype; this decision should be take also in consideration other parameters, including disease severity, tolerability, adherence to the therapy, and comorbidities.

## Conclusions and perspectives

Several independent studies have clearly shown a close association of *IL-28B* SNPs with treatment response to PEG-IFN plus RBV, with consistent results among patients of different ethnic origin. Predictive models have been developed that attempt to incorporate *IL28B* genotyping into clinical decision-making. This will open a window for genotype-based personalized medicine for patients with chronic hepatitis C. However, genetic data about HCV patients are far from being complete and further investigations, based either on GWAS technology improvement or candidate gene approach, are necessary for an exhaustive “genetic profiling” of patients chronically infected with HCV. In fact, we should keep in mind that treatment response is currently predicted by many factors likely to be unrelated to *IL-28B* SNPs, such as age, gender, viral genotype, fibrosis and compliance.

## Abbreviations

GWAS: genome-wide association studies; SNP: single nucleotide polymorphism; IL28B: interleukin 28B; HCV: Hepatitis C virus; PEG-IFN: pegylated interferon; SRV: sustained viral response; Jak-STAT: Janus kinase - Signal Transducer and Activator of Transcription.

## Competing interests

The authors declare that they have no competing interests.

## Declarations

Publication of this supplement was partly supported by an unrestricted grant provided by Roche. The articles were independently prepared by the authors with no input from Roche. Roche were not involved in selecting the articles for the supplement. The PEG-IFN-alpha treatment mentioned in this article is produced by Roche.
